# Spatial Forecast of Landslides in Three Gorges Based On Spatial Data Mining

**DOI:** 10.3390/s90302035

**Published:** 2009-03-18

**Authors:** Xianmin Wang, Ruiqing Niu

**Affiliations:** Institute of Geophysics and Geomatics, China University of Geosciences / No. 388 Lumo Road, Wuhan, P.R. China; E-mail: rqniu@163.com (R.N.)

**Keywords:** Remote sensing image, landslide, forecast, Three Gorges

## Abstract

The Three Gorges is a region with a very high landslide distribution density and a concentrated population. In Three Gorges there are often landslide disasters, and the potential risk of landslides is tremendous. In this paper, focusing on Three Gorges, which has a complicated landform, spatial forecasting of landslides is studied by establishing 20 forecast factors (spectra, texture, vegetation coverage, water level of reservoir, slope structure, engineering rock group, elevation, slope, aspect, etc). China-Brazil Earth Resources Satellite (Cbers) images were adopted based on C4.5 decision tree to mine spatial forecast landslide criteria in Guojiaba Town (Zhigui County) in Three Gorges and based on this knowledge, perform intelligent spatial landslide forecasts for Guojiaba Town. All landslides lie in the dangerous and unstable regions, so the forecast result is good. The method proposed in the paper is compared with seven other methods: IsoData, K-Means, Mahalanobis Distance, Maximum Likelihood, Minimum Distance, Parallelepiped and Information Content Model. The experimental results show that the method proposed in this paper has a high forecast precision, noticeably higher than that of the other seven methods.

## 1. Introduction

The Three Gorges is an area in China in which the distribution density of landslides is very high and the population is very concentrated. In Three Gorges there are frequent landslides and the potential risk of landslide disasters is enormous. After the water has been normally sluiced in the Three Gorges dam, under the effect of factors such as high water levels, water level changes in the reservoir and rainfall, the landslides obviously become more active. It is expected that for a decade or maybe even up to twenty years or more after the Three Gorges Dam has been built, these landslides would be still very active, so if landslides are not studied, spatially forecasted, prevented and remedied, this could have a large and detrimental influence on the normal operation of the dam, navigation buildings and workshops in Three Gorges, which could result in the reservoir in Three Gorges not being able to be normally sluiced and even endanger the properties and lives of the nearby inhabitants.

Extensive attention has been paid internationally to the study of landslide risks. The gestation and occurrence of landslides is greatly influenced by various factors and it is often quite difficult to establish the causality between the occurrence of landslides and those factors. The restriction of the evaluation methods to single factor quantification has a direct influence on the reliability and veracity of the evaluation results, therefore those methods are largely restricted to the area of spatial forecasting of regional landslides. At present the key techniques and methods for effective and scientific spatial forecast of regional landslides are both hot research topics and challenges in the area of landslide forecasting.

In the past 30 years, national and international researchers have conducted many studies on spatial forecasting of landslide disasters [1–4]. This research has mostly addressed two aspects [1,3]: establishment of semi-quantitative or quantitative sensitivity indexes for landslide disasters by comparing the distribution maps of landslide disasters with the maps of various factors, and secondly calculation and analysis of the sensitivity indexes, and adoption of the high and the low sensitivity indexes to represent the dangerous and stable regions, respectively, of landslide disasters. The other is to theoretically analyze the relationships between landslided and various influencing factors, adoption of methods of scoring or appraising factors to assign weights to the various factors, and then calculate the weight coefficients to establish the risk levels for landslides. At present, the major forecasting methods include experimental models (expert scoring), symbolic statistic models (regress analysis, judgement analysis, clustering analysis), information models, fuzzy judegement models (fuzzy synthetical judgement, fuzzy reliability analysis), gray models, pattern recognition models (expert system, neural network), nonlinear models (Fractal theory), and so on, of which the Information Content Model is the more mature and the one that is applied more extensively. Along with high-speed development of computer technique and geoinformatics, the combination of GIS (Geographical Information Science) and quantitative spatial forecast methods for landslides has become a new area in geological disaster research.

Yin *et al.* [5,6] and Yan *et al*. [7] have carried out deep and systematic research on spatial forecasting and the stability partitioning of landslides and slopes and they have proposed models such as the Information Analysis Model [5], the Multi-factor Regress Model [6], the Clustering Analysis Model [7] and the Judgement Analysis Model [7]. However the precision of these models is not high (about 75–80%). Xie *et al*. [8] adopted the Information Content Model to make spatial forecasts of landslides in Pangan County, Zhejiang Province, in August, 2004 when Typhoon Yunna came to Zhejiang Province, and the forecast precision reached 75.8%. Zhu *et al*. [9] adopted Information Content Model to segment landslide risk districts in the upriver regions of the Yangtse, Chongqing Province, Sichuang Province, Guizhou Province. Based on landslide hazard analysis they utilized the MapGIS software platform to perform a fragility analysis of the regional economy and carry out landslide risk assessments. Shan *et al*. [10] adopted Artificial Neural Network and GIS, combined nonlinear theory and spatial multi-analysis and established a nonlinear forecast model of landslides based on environmental factors in the middle region of Da Yu Island in Hongkong. Recently international scholars began to research the relationship between single environmental factors and landslides in a region. Lumb [11] analyzed the pertinence of different types of deposits, underlying bed rock and landslides. The recurrence of slope failures in residual soils of Hong Kong is analysed for the period 1950 to 1973, and various factors contributing to the instability are described. It is postulated that the prime cause of the failures is direct infiltration of rain water into the surface zones of the slopes, producing a loss of effective cohesion following the saturation of the soil. Prevention of slips implies protection against excessive infiltration. Ruxton [12] analyzed the relationship between mantle rock and landslides and discovered efflorescence had an important influence on landslides. Fourie [13] studied the relationship between rainfall pervasion and shallow landslips. He found slope failures usually occurred in regions of the world where steep slopes consisting of residual soils were subjected to periods of prolonged and heavy rainfall. A mechanism for the failure of these slopes was postulated whereby *in situ* soil suctions were decreased by the ingress of a wetting front until a critical depth was reached where the shear strength of the soil was no longer sufficient to ensure stability. He proposed a technique for predicting whether a particular rainfall event (defined in terms of intensity, duration and return period) would cause ingress of a wetting front to this critical depth, with particular reference to the failure of a number of road embankments in the Northern Province of South Africa. Collison [14] studied the relationship between vegetation and slope stability in torrid zones and discovered that along with growth of vegetation roots, water permeability would increase and soil strength would decrease. Evans [15] studied the relationship between elevation and rainfall. He found the underlying geology and the angle of slope were the most important parameters for determining natural terrain landslide susceptibility on a regional scale. Geological strata which appeared to be particularly susceptible included rhyolitic and dacitic lavas, jointed tuffs, layered sequences of volcaniclastic rocks and lavas, and layered sedimentary sequences. The most susceptible slopes were generally those with angles of approximately 35° to 40°. The shape and aspect of a particular slope may also be useful in assessing susceptibility. Carrara [16] utilized 1,500 landslides in the GIS database and studied the relationship between shallow landslides and landforms, and the results showed that there existed a good statistical correlation between abrupt landforms and landslides. Brabb [17] took slope as the weight, calculated the percentages of 2,000 landslides and 12 factors and concluded geology, soil and slope are the major factors which have an influence on landslide stability.

Traditional research on landslides lacks information extraction and mining of complicated landslide disaster systems and appropriate consideration of the indeterminacy and nonlinear character of landslide systems, and forecasting models based on a single factor cannot produce exact forecasts and estimations of landslide disasters. Furthermore the data come from a variety of sources, and as the means of data accumulation are improved, the contents of the forecast database for landslide disasters have become enormous and more complicated, so the current trend is to determine how to adequately utilize the large amount of available data to realize the spatial forecasting of landslides and increase the precision and effectiveness of forecasts [18–22]. It should be possible to directly drive the development of the theory and technique of landslide risk assessment by utilizing artificial intelligence techniques based on spatial data mining and knowledge discovery. However, at present scholars and researchers primarily use the traditional methods to carry out spatial forecasts of landslides, which need manual intervention, so they are characterized by poor intelligence, low precision and effectiveness. Zhao *et al*. [23] adopted a decision tree to estimate landslide risk, but he only chose 4 factors of lithology, elevation, slope and spectra, and in his forecast result, many landslides fell in the low risk region and the precision isn’t high. Ma *et al*. [24] adopted a support vector machine to forecast and assess landslide disasters, but his forecast precision is merely 75.45%, and in his results, there were still some landslides lying in stable regions.

In the paper, and considering the Three Gorges, with a complicated landform and frequent landslide disasters, spatial forecast of landslides is studied by establishing 20 forecast factors of spectra, texture, vegetation coverage, water level of reservoir, slope structure, engineering rock group, elevation, slope, aspect, etc. China-Brazil Earth Resources Satellite (Cbers) images, geological maps and terrain maps are adopted based on a C4.5 decision tree to mine spatial forecast criteria for landslides in Guojiaba Town, Zhigui County, in the Three Gorges region and, based on this knowledge perform intelligent spatial forecast of landslides in Guojiaba Town. All landslides lie in the dangerous and unstable regions, so the forecast result is good and the precision is high.

## 2. Choice of Forecast Factors

The nature and severity of landslide disasters are related to many factors such as geological structure, stratum, lithology, terrain and physiognomy, vegetation coverage, rainfall, human and engineering activities. In this paper, the characteristics and factors influencing landslides in the Three Gorges region are analyzed; 20 forecast factors are established referring to the aspects of spectra, texture, vegetation coverage, water level of reservoir, slope structure, engineering rock group, elevation, slope and aspect.

### 2.1. Spectral Factors

Remote sensing images can factually reflect ground circumstances and information, and it has become a new research trend to adopt remote sensing images to perform quantitative detection and forecast of landslide disasters [25–28]. In this paper Cbers images of 19.5 metres resolution produced in Three Gorges in 2006 were adopted to study the spatial forecasting of landslides. Division between two spectra can effectively restrain some interferential information and give prominence to the key information. To effectively restrain vegetation information and give prominence to lithology information, the division between the two spectra of Cbers3 and Cbers2 is made. The forecast spectral factors are chosen as Cbers1, Cbers2, Cbers3, Cbers4 and Cbers3/Cbers2.

### 2.2. Textural Factors

**Figure 1.**
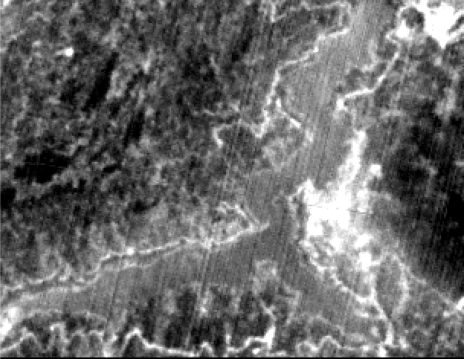
Cbers1 image of Guojiaba Town in Three Gorges.

**Figure 2.**
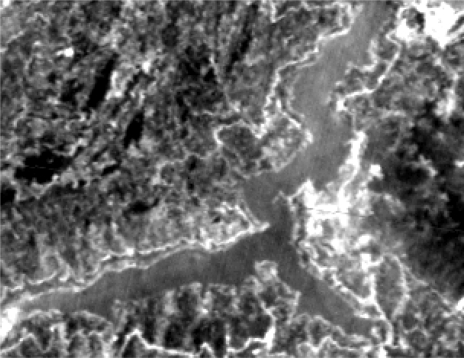
Cbers2 image of Guojiaba Town in Three Gorges.

**Figure 3.**
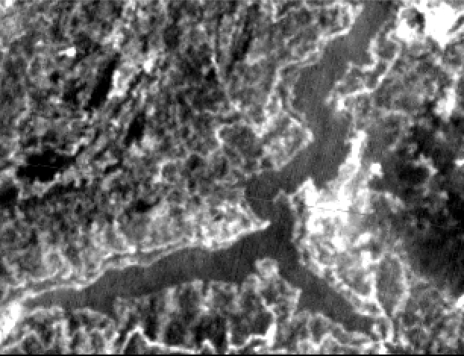
Cbers3 image of Guojiaba Town in Three Gorges.

**Figure 4.**
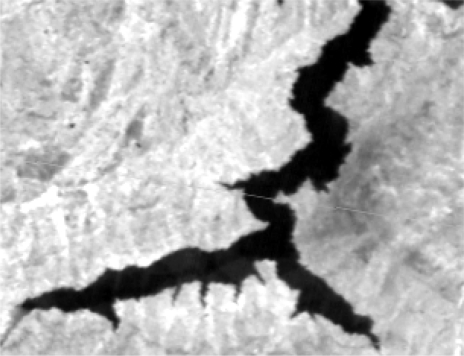
Cbers4 image of Guojiaba Town in Three Gorges.

The form of landslides is connected the strata and lithology. The texture is a special feature of some strata and lithology and can be directly used to analyze and recognize landslides [29–32]. The four Cbers spectral images are shown in Figures 1–4, respectively. In Cbers3 the textural information is the most clear and abundant, so the textural information in Cbers3 image is chosen to recognize and forecast landslides.

The Gray level co-occurrence matrix (GLCM) describes the features of the spatial distribution and structure of various pixels in the image and possesses the prominence to improve recognition and interpretation of geoscience objects by remote sensing images. GLCM provides many textural factors which describe the textural features of the image from different aspects. In the paper, 8 textural factors of GLCM are chosen to recognize and forecast the landslide.

1. Contrast
  (1) $$\mathrm{CON}=\sum_{i=1}^{n}\sum_{j=1}^{n}p{\left(i,j\right)}^{2}\times {\left(i-j\right)}^{2}$$
2. Correlation
  (2) $$\mathrm{COR}=[\sum_{i=1}^{n}\sum_{j=1}^{n}p(i,j)\times (i-n)\times (j-n)]/(\sqrt{i-n^{2}}\times \sqrt{j-n^{2}})$$
3. Mean
  (3) $$\mathrm{MEAN}=\sum_{i=1}^{n}\sum_{j=1}^{n}\left(i-1)\times p(i,j\right)$$
4. Entropy
  (4) $$\mathrm{ENT}=-\sum_{i=1}^{n}\sum_{j=1}^{n}p(i,j)\times {\text{lg}}^{p(i,j)}$$
5. Homogeneity
  (5) $$\mathrm{HOMO}=\sum_{i=1}^{n}\sum_{j=1}^{n}\frac{p(i,j)}{1+{\left(i-j\right)}^{2}}$$
6. Dissimilarity
  (6) $$\mathrm{DIS}=\sum_{i=1}^{n}\sum_{j=1}^{n}\left|i-j\right|p(i,j)$$
7. Angle Second Moment
  (7) $$\mathrm{ASM}=\sum_{i=1}^{n}\sum_{j=1}^{n}\sqrt{0.5\left|i-j\right|p(i,j)}$$
8. Variance
  (8) $$\mathrm{Variance}=\sum_{i=1}^{n}\sum_{j=1}^{n}{\left(i-\mathrm{Mean}\right)}^{2}\times p(i,j)$$
  in which *p*(*i, j*) is the pixel value in the position (*i, j*) in GLCM.

### 2.3. Vegetation Coverage Factors

In Three Gorges there is flourishing vegetation and on different strata and rocks, the types and degrees of vegetation growth are also different. Landslides often happen in strata with low vegetation coverage. For the regions of high vegetation coverage, the vegetation index is an effective forecast factor. Vegetation index is a quantitative value extracted from a multi-spectral remote sensing image and reflects the vegetation condition on the surface of the earth. Normalized Difference Vegetation Index (NDVI) possesses a wide detection range of vegetation coverage and good adaptability of time phase and space, so NDVI is chosen as the vegetation coverage forecast factor.

### 2.4. Geological, Physiognomy and Environmental Factors

According to 1:0.05 million geological map and 1:0.01 million terrain map, six geological structure, physiognomy and environmental forecast factors are established, such as water level of the reservoir, slope structure, engineering rock group, elevation, slope and aspect. The slope structure and engineering rock group factors are provided by the geological map and the water level of the reservoir factor is provided by the terrain map. The contour line data are protracted by an aerial survey performed in Nov. 2007 and provided by the Three Gorges Headquarters. The factors of elevation, slope and aspect are produced from contour data. According to the influence of water fluctuation, the water level of the reservoir is classified into four classifications: poorly influenced region, secondly influenced region, strongly influenced region and fluctuating region. Slope structure is classified into five classifications: slopes with converse direction, slopes with the same direction, slopes with horizontal direction, converse slope and direct slope. Engineering rock group is classified into four classes: soft rock, hard rock, alternate soft and hard stratum, loose deposits.

## 3. Decision Tree C4.5 Algorithm

C4.5 algorithm [20] based on ID3 algorithm adds the function of translating a decision tree into equivalent production rules and solving the continue value study problem. C4.5 adopts an information entropy method and chooses the attribute of maximum information gain rate and the corresponding segment threshold as the best test attribute and segment threshold.

### 3.1. Production of Decision Tree

1. Calculating information entropy in classification

Suppose *S* be the number of samples in training sets and there is *m* classifications of samples *C_i_* (*i*=1, 2, …, *m*). *S_i_* is the number of samples in Classification *C_i_*. The computational formula is as follows:

(9) $$I(S_{1},S_{2},\cdots ,S_{m})=-\sum_{i=1}^{m}p_{i}\;{\text{log}}_{2}\;(p_{i})$$

in which *p_i_* = *S_i_/S* is the probability of arbitrary sample belonging to *C_i_*.

2. Calculating information entropy of each attribute

Suppose Attribute *X* possesses *v* values {*x*_1_, *x*_2_, …, *x*_v_}, which divides *S* into *v* subsets {*s*_1_, *s*_2_, …, *s_v_*}. *S_j_* includes those samples in *S* which take the value *x_j_* on Attribute *X* (*j*=1, 2, …, *v*). The Expect Entropy (Condition Entropy) of taking Attribute *X* as the classification attribute is:

(10) $$E(X)=\sum_{j=1}^{v}\frac{s_{1j}+\cdots s_{\mathrm{mj}}}{s}I(s_{1j},\cdots ,s_{\mathrm{mj}})$$

in which *s_ij_* is the number of samples which belong to Classification *C_i_* in Subset *s_j_*, and $I(s_{1j},s_{2j},\cdots ,s_{\mathrm{mj}})=-\sum_{i=1}^{m}p_{\mathrm{ij}}{\text{log}}_{2}(p_{\mathrm{ij}})$, in which $p_{\mathrm{ij}}=\frac{s_{\mathrm{ij}}}{s_{j}}$ is the probability of each sample in *s_j_* belonging to *C_i_*.

3. Calculating information gain and information gain rate of attribute

The information gain function of Attribute *X* is:

(11) $$\mathrm{Gain}(X)=I(S_{1},S_{2},\cdots ,S_{m})-E(X)$$

The information gain function tends to produce a big value for the test, which probably produces multi-branches. However a test of producing multi-branches doesn’t mean it can obtain a better forecast result for those unknown objects. The information gain rate function can make up the lack of information gain. Information gain rate is an improvement of information gain which can eliminate the influence of the attribute of producing multi-branches. The information gain function considers not only the number of nodes but also the size of each node (number of samples included) for each segment. What it considers is not the amount of information included in classifications but each segment. The information gain rate of Attribute *X* is:

(12) $$A(X)=\frac{\mathrm{Gain}(X)}{I(S_{1},S_{2},\cdots S_{v})}$$

in which *v* is the number of branches of the node and *S_i_* is the number of records for the *i*th branch.

4. Producing decision tree

It in turn calculates the information gain *Gain*(*X*) and information gain rate *A*(*X*) of each attribute and chooses the as the test attribute the one which possesses the biggest information gain rate and the information gain value that is not lower than the average of the information gains of all the attributes. It takes the test attribute as a node and each distribution of the attribute as a branch to segment the samples. If all the samples of a node belong to the same class, the node is a leaf which is marked by its classification. It would form the initial decision tree by recursion when all the samples of each subset obtain the same value on the main attribute or there is no attribute for being utilized.

### 3.2. Pruning of Decision Tree

In order to remove singular branches introduced by noise data and isolated points, a pruning of the initial decision tree by a pruning algorithm should be done. Firstly, for each non-leaf node it calculates Expect Error Probability while the sub-tree of the node is clipped. Secondly it utilized the error rate of each branch combined with the weight of each branch to calculate the Expect Error Rate while not clipping the branch. If Expect Error Rate while clipping the branch obtains higher value than the one while not clipping the branch, the sub-tree is retained. Otherwise the sub-tree is clipped. Finally it would obtain the decision tree of the smallest Expect Error Rate.

## 4. Experiments of Criterion Mining and Spatial Intelligent Forecast of Landslides in Three Gorges

**Figure 5.**
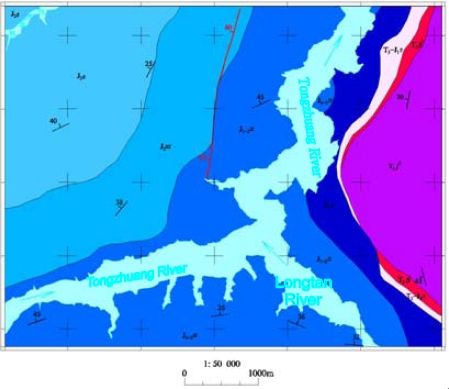
1: 0.05 million geological map of Guojiaba.

In the paper Guojiaba Town Zhigui County in Three Gorges was chosen as a research area in which landslides happen frequently. Guojiaba Town is composed of the strata of Upper Shaximiao Group J_2_s, Lower Shaximiao Group J_2_xs, Niejiashan Group J_1–2_n, Xiangxi Group J_1_x, Shazhenxi Group T_3_-J_1_s, Ba First Section Badong Group T_2_b^1^ and Jia Third Section Jialingjiang Group T_1_j^3^. In Guojiaba Town the geological structure is very complicated and there are more than 30 landslides distributed there, for example, Shizibao Landslide, Jinchaiwan Landslide, Zhangjiawan Landslide, North Longwangmiao Landslide, Longtanwan Landslide, Dengjiapo Landslide and South Cement Factory Landslide. A 1:0.05 million geology map of Guojiaba Town is shown in Figure 5.

In the paper Cbers imagea with 19.5 resolution produced in 2006 were adopted. They are shown in Figures 6–7. The piling graph of the remote sensing image with the disaster distribution graph is shown in Figure 8. The primarily adopted forecast factor images are shown in Figures 9–17.

**Figure 18.**
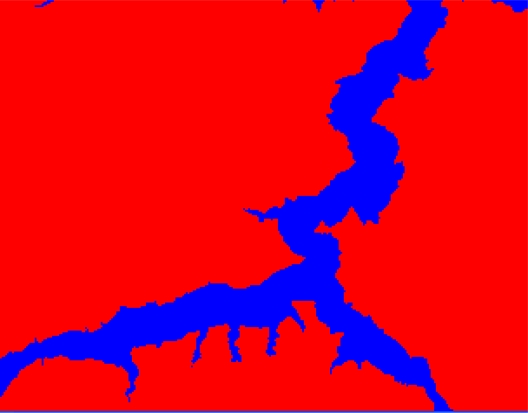
Forecast result of IsoData method.

**Figure 19.**
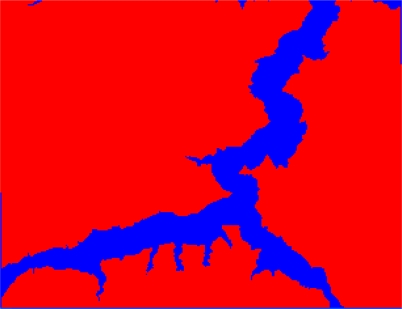
Forecast result of K—Means method.

**Figure 20.**
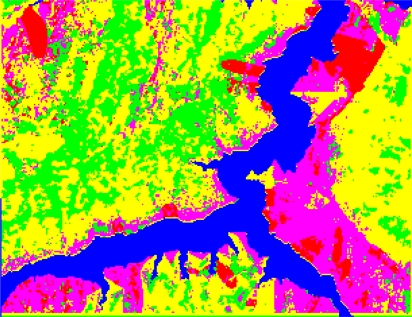
Forecast result of Mahalanobis Distance.

**Figure 21.**
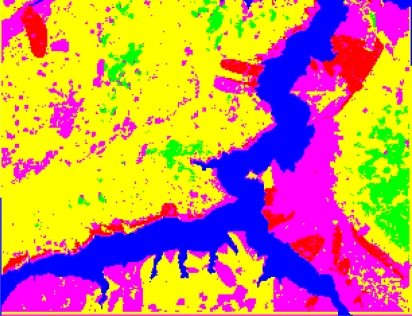
Forecast result of Maximum likelihood.

**Figure 22.**
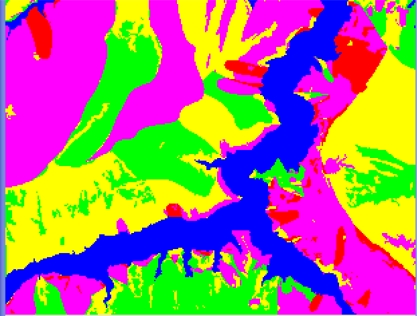
Forecast result of Information Content Model.

**Figure 23.**
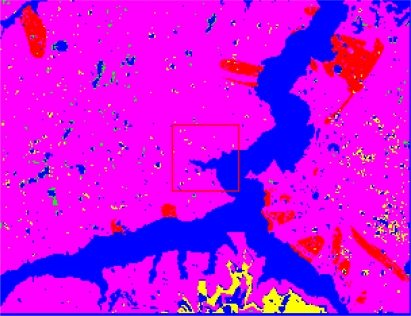
Forecast result of Parallelepiped.

**Figure 24.**
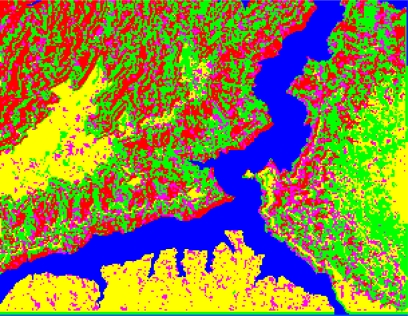
Forecast result of Minimum Distance.

**Figure 25.**
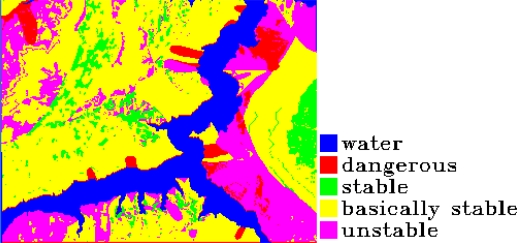
Forecast result of Decision Tree Mehod.

In the paper 2,696 sample points are chosen to produce the decision tree of 167 leaf nodes, and the study precision is 99.5%. The decision tree after pruning includes 136 leaf nodes and the study precision is 99.1%, which is shown in Table 1. The mined criteria of spatial forecast of landslides in Guojiaba Town are shown in Table 2. The rules possess high confidence values and the precision of rule extraction is 99.3%. The connotations of the values of some forecast indexes are shown in Table 3. Spatial forecast of landslides in Guojiaba Town Zhigui County in Three Gorges was done based on knowledge driving, and the forecast result is compared with the ones obtained by seven methods: Information Content Model, IsoData Method, K-Means Method, Mahalanobis Distance, Maximum likelihood, Minimum Distance and Parallelepiped. The forecast precision of decision tree method is 99.15%, obviously superior to the other seven ones. The forecast results and precisions of various methods are shown respectively in Figures 18–25 and Table 4.

The experimental results have shown that IsoData Method and K-Means Method define all the pixels as a dangerous region, and Parallelepiped Method divides most of pixels into dangerous region and unstable region, which is obviously inappropriate and gives low forecast precision. The Minimum Distance Method is not good at dividing dangerous region, unstable region and stable region and the forecast precision is also low. The Information Content Model erronueously assigns the basically stable area to the north of Guojiaba Town as an unstable region. The Maximum Likelihood Method and Mahalanobis Distance Method can distinguish four kinds of regions as dangerous, unstable, basically stable and stable ones, but they still make mistakes in some areas and their precisions are average. The Decision Tree Method does well in the spatial forecast of landslides and obtains a very high forecast precision.

**Figure 26.**
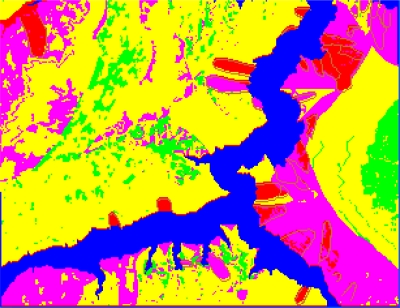
Forecast results of decision tree added to the disaster distribution map.

The forecast result of Decision Tree Method is added to the disaster distribution map, which is shown in Figure 26. The compiled result shows that all the landslides in Guojiaba Town lie in dangerous and unstable regions and confirm that the Decision Tree Method provides a good forecast result and a very high forecast precision which is obviously superior to the other seven methods.

## 5. Conclusions

In Three Gorges there are extensively distributed active landslides and the population is very concentrated, so the potential landslide risk is tremendous. To guarantee the normal running of the dam, buildings for navigation and workshops and to safeguard the properties and lives of the inhabitants in Three Gorges, the landslides should be exactly predicted and forecasted, so it is very important and significant to make accurate spatial forecast of landslides in the Three Gorges region.

The formaton of landslides is related to many factors of geological structure, strata, lithology, terrain and physiognomy, vegetation coverage, rainfall, human and engineering activities. By studying the mechanisms of landslides in Three Gorges, 20 forecast factors are established covering the various aspects of spectra, texture, vegetation coverage, water level of reservoir, slope structure, engineering rock group, elevation, slope and aspect. Those factors are closely related to the mechanism and formation of landslides in Three Gorges.

China-Brazil Earth Resources Satellite (Cbers) image with the resolution of 19.5 meters can factually reflect ground circumstances and does well in monitoring and forecasting regional landslides. Cbers images produced in 2006 were adopted to spatially forecast landslides in Three Gorges.

The Decision Tree Method is the most attractive data mining method and does well in pattern recognition and classification. The C4.5 algorithm adopts an information entropy method and chooses the attribute of maximum information gain rate and the corresponding segment threshold as the best test attribute and segment threshold. In this paper Guojiaba Town (Zhigui County) in the Three Gorges region, in which there are more than 30 landslides, was chosen as the study area. The C4.5 algorithm was adopted to produce the decision tree after pruning with the study precision 99.1% and mine the spatial forecast criterions of landslides in Guojiaba Town. The mined criteria possess high confidence values and the precision of criteria extraction is 99.3%.

Based on the mined criteria by knowledge driving intelligent spatial forecast of landslides in Guojiaba Town is realized, with the high forecast precision (99.15%) and Kappa Coefficient (0.9876). All landslides lie in the dangerous and unstable regions, so the forecast result is good. Another seven methods were also adopted to make spatial forecasts of landslides in Guojiaba Town, which are IsoData, K-Means, Mahalanobis Distance, Maximum likelihood, Minimum Distance, Parallelepiped and Information Content Model. The forecast precisions of the seven methods are 15.99%, 15.99%, 73.44%, 80.97%, 28.21% and 46.59%, respectively, with Kappa Coefficients of 0, 0, 0.6311, 0.7322, 0.0906 and 0.2126. By the seven methods some landslides are even classified as stable regions, or the stable and basically stable regions are mistakenly recognized as dangerous or unstable ones. The method proposed in the paper can realize accurate spatial forecast of landslides and is obviously superior to the other seven ones tested. Furthermore in the paper the mined forecast criteria possess the virtues of quantification, so they can provide intelligent spatial forecast and interpretation of landslides in the important Three Gorges region.

## Figures and Tables

**Figure 6.**
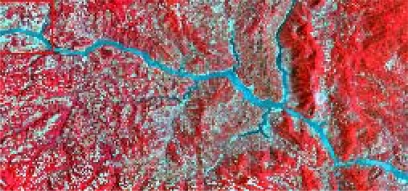
Cbers image composed of 432 spectra in Three Gorges.

**Figure 7.**
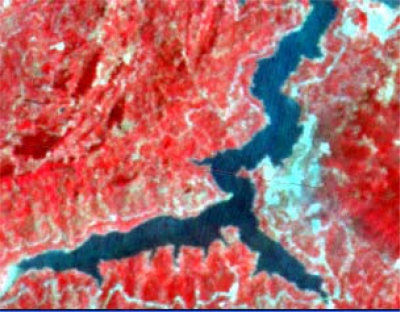
Cbers image composed of 432 spectra in Guojiaba Town.

**Figure 8.**
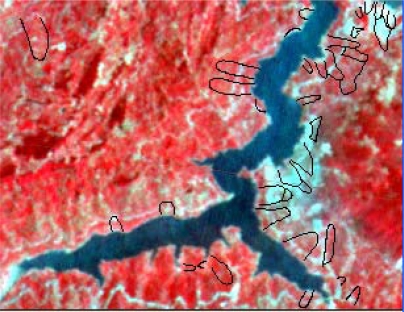
Cbers image piled up with landslide disaster distribution graph.

**Figure 9.**
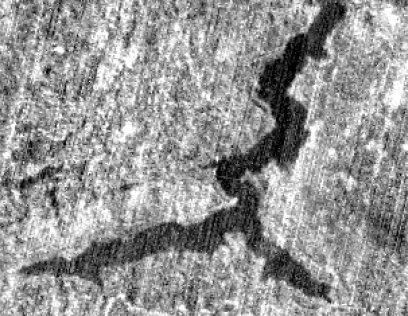
Cbers3/Cbers2 image.

**Figure 10.**
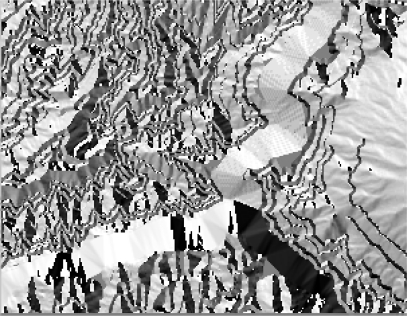
Aspect image.

**Figure 11.**
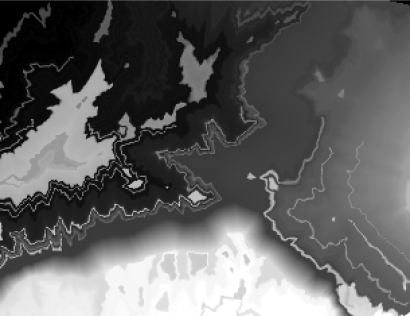
DEM image.

**Figure 12.**
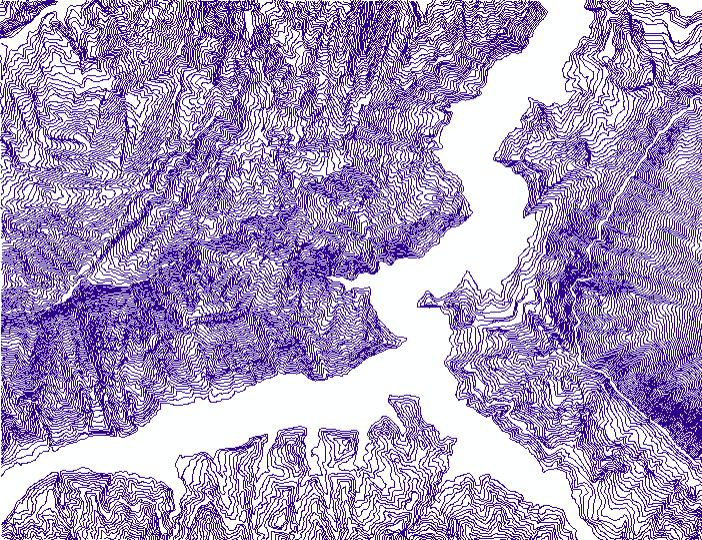
Contour.

**Figure 13.**
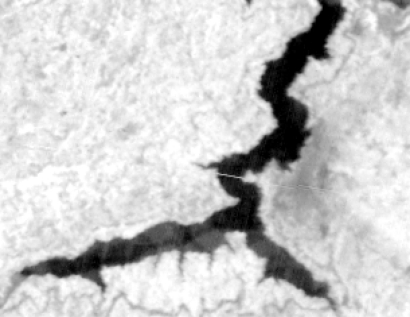
NDVI image.

**Figure 14.**
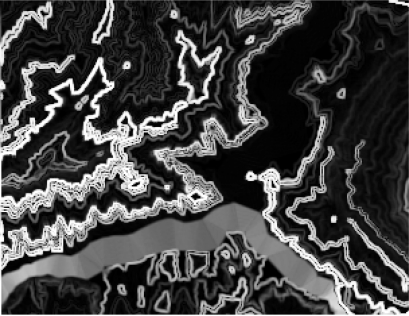
Slope image.

**Figure 15.**
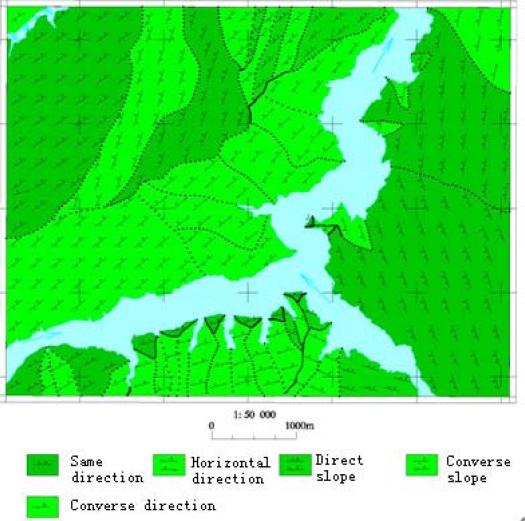
Slope structure image.

**Figure 16.**
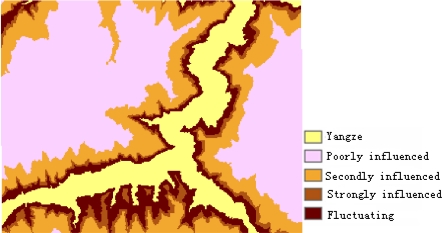
Reservoir water level.

**Figure 17.**
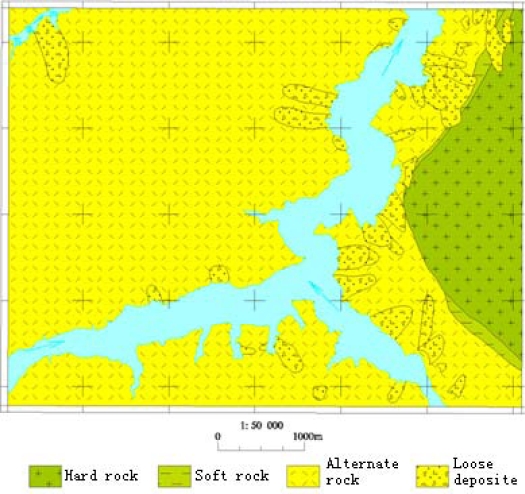
Distribution graph of engineering rock group.

**Table 1.** Decision tree after pruning.

engineering rock group = 0.0000: dangerous (3.0/1.1)
engineering rock group = 1.6000: unstable (26.0/1.3)
engineering rock group = 1.2000:
| slope structure = 0.0000: basically stable (0.0)
| slope structure = 0.9000: basically stable (66.0/1.4)
| slope structure = 1.2000: basically stable (0.0)
| slope structure = 0.6000:
| | band3dissimilarity <= 4.2222 :
| | | band3variance <= 11.8765 : basically stable (340.0/3.9)
| | | band3variance > 11.8765 :
| | | | water level of reservoir = 0.1000: stable (9.0/2.4)
| | | | water level of reservoir = 0.2000: basically stable (2.0/1.0)
| | | | water level of reservoir = 0.4000: stable (0.0)
| | | | water level of reservoir = 0.5000: basically stable (2.0/1.0)
| | | | water level of reservoir = 0.0000: stable (0.0)
| | band3dissimilarity > 4.2222 :
| | | water level of reservoir = 0.1000: stable (52.0/1.4)
| | | water level of reservoir = 0.2000: basically stable (7.0/1.3)
| | | water level of reservoir = 0.4000: basically stable (2.0/1.0)
| | | water level of reservoir = 0.5000: basically stable (1.0/0.8)
| | | water level of reservoir = 0.0000: stable (0.0)
| slope structure = 1.5000:
| | band2 <= 77 : basically stable (36.0/1.4)
| | band2 > 77 :
| | | water level of reservoir = 0.4000: unstable (23.0/1.3)
| | | water level of reservoir = 0.5000: unstable (0.0)
| | | water level of reservoir = 0.0000: unstable (0.0)
| | | water level of reservoir = 0.1000:
| | | | dem > 139 : unstable (15.0/1.3)
| | | | dem <= 139 :
| | | | | dem <= 41 : unstable (2.0/1.0)
| | | | | dem > 41 : basically stable (59.0/1.4)
| | | water level of reservoir = 0.2000:
| | | | band4 <= 150 :
| | | | | band3variance <= 8.1728 : unstable (387.0/1.4)
| | | | | band3variance > 8.1728 :
| | | | | | dem <= 78 : basically stable (4.0/1.2)
| | | | | | dem > 78 : unstable (50.0/2.6)
| | | | band4 > 150 :
| | | | | dem <= 126 : basically stable (4.0/1.2)
| | | | | dem > 126 : unstable (10.0/1.3)
| slope structure = 0.3000:
| | NDVI <= 0.2917 : unstable (38.0/1.4)
| | NDVI > 0.2917 :
| | | band4 <= 158 :
| | | | band3 <= 37 :
| | | | | dem <= 337 : stable (13.0/1.3)
| | | | | dem > 337 : basically stable (18.0/1.3)
| | | | band3 > 37 :
| | | | | aspect <= 298.811 :
| | | | | | band4 <= 155 : basically stable (90.0/1.4)
| | | | | | band4 > 155 :
| | | | | | | slope <= 39.0801 : basically stable (18.0/1.3)
| | | | | | | slope > 39.0801 : stable (2.0/1.0)
| | | | | aspect > 298.811 :
| | | | | | band4 <= 151 : basically stable (7.0/1.3)
| | | | | | band4 > 151 : unstable (3.0/1.1)
| | | band4 > 158 :
| | | | slope > 10.1868 : stable (71.0/1.4)
| | | | slope <= 10.1868 :
| | | | | aspect <= 276.87 : basically stable (6.0/1.2)
| | | | | aspect > 276.87 : unstable (2.0/1.8)
engineering rock group = 2.0000:
| slope structure = 0.6000: dangerous (22.0/1.3)
| slope structure = 0.0000: unstable (0.0)
| slope structure = 0.3000: unstable (305.0/1.4)
| slope structure = 0.9000: dangerous (39.0/1.4)
| slope structure = 1.2000: dangerous (11.0/1.3)
| slope structure = 1.5000:
| | dem > 362 : unstable (25.0/1.3)
| | dem <= 362 :
| | | aspect <= 259.38 :
| | | | dem <= 147 : dangerous (53.0/1.4)
| | | | dem > 147 :
| | | | | band3 <= 46 : dangerous (19.0/2.5)
| | | | | band3 > 46 :
| | | | | | aspect <= 197.526 :
| | | | | | | band3homogeneity > 0.5222 : dangerous (19.0/1.3)
| | | | | | | band3homogeneity <= 0.5222 :
| | | | | | | | band3correlation > −3.0981 : unstable (9.0/1.3)
| | | | | | | | band3correlation <= −3.0981 :
| | | | | | | | | dem <= 161 : unstable (6.0/1.2)
| | | | | | | | | dem > 161 :
| | | | | | | | | | band4 > 145 : unstable (6.0/1.2)
| | | | | | | | | | band4 <= 145 :
| | | | | | | | | | | band3mean <= 19.6667 : dangerous (44.0/7.2)
| | | | | | | | | | | band3mean > 19.6667 :[S1]
| | | | | | aspect > 197.526 :
| | | | | | | band4 > 128 : unstable (58.0/2.6)
| | | | | | | band4 <= 128 :
| | | | | | | | water level of reservoir = 0.1000: unstable (1.0/0.8)
| | | | | | | | water level of reservoir = 0.4000: dangerous (4.0/1.2)
| | | | | | | | water level of reservoir = 0.5000: unstable (0.0)
| | | | | | | | water level of reservoir = 0.0000: unstable (0.0)
| | | | | | | | water level of reservoir = 0.2000:
| | | | | | | | | band3mean <= 20.7778 :
| | | | | | | | | | dem <= 187 : unstable (4.0/1.2)
| | | | | | | | | | dem > 187 : dangerous (8.0/1.3)
| | | | | | | | | band3mean > 20.7778 :
| | | | | | | | | | band4 > 118 : unstable (18.0/1.3)
| | | | | | | | | | band4 <= 118 :
| | | | | | | | | | | band3mean <= 23.6667 : dangerous (7.0/1.3)
| | | | | | | | | | | band3mean > 23.6667 : unstable (2.0/1.0)
| | | aspect > 259.38 :
| | | | band4 <= 152 : dangerous (195.0/7.3)
| | | | band4 > 152 :
| | | | | band3entropy <= 2.0432 : dangerous (2.0/1.0)
| | | | | band3entropy > 2.0432 : unstable (5.0/1.2)
engineering rock group = 0.4000:
| slope structure = 0.6000: basically stable (0.0)
| slope structure = 0.0000: basically stable (0.0)
| slope structure = 0.3000: unstable (14.0/1.3)
| slope structure = 0.9000: basically stable (0.0)
| slope structure = 1.2000: basically stable (0.0)
| slope structure = 1.5000:
| | dem <= 263 : basically stable (231.0/1.4)
| | dem > 263 :
| | | band3variance > 1.284 : stable (73.0/1.4)
| | | band3variance <= 1.284 :
| | | | aspect <= 257.735 :
| | | | | slope <= 3.7242 :
| | | | | | band3secondmoment <= 0.1852 : stable (8.0/1.3)
| | | | | | band3secondmoment > 0.1852 : basically stable (3.0/2.1)
| | | | | slope > 3.7242 :
| | | | | | band4 <= 128 : stable (2.0/1.0)
| | | | | | band4 > 128 : basically stable (68.0/3.8)
| | | | aspect > 257.735 :
| | | | | 3/2 <= 0.4949 : basically stable (3.0/2.1)
| | | | | 3/2 > 0.4949 : stable (29.0/1.4)
Subtree [S1]
band3contrast > 19.2222 : dangerous (6.0/1.2)
band3contrast <= 19.2222 :
| band3homogeneity <= 0.3951 : unstable (16.0/2.5)
| band3homogeneity > 0.3951 :
| | band3contrast <= 3.8889 : unstable (7.0/2.4)
| | band3contrast > 3.8889 : dangerous (6.0/1.2)

**Table 2.** Forecast criteria for landslide.

Forecast criterions of stable region:
Rule 1: band3variance > 1.284 & engineering rock group = 0.4000 & dem > 263 -> class stable [98.1%]
Rule 2: band4 > 158 & slope > 10.1868 & engineering rock group = 1.2000 & slope structure = 0.3000 -> class stable [98.1%]
Rule 3: 3/2 > 0.4949 & engineering rock group = 0.4000 & aspect > 257.735 & dem > 263 -> class stable [98.0%]
Rule 4: water level of reservoir = 0.1000 & band3dissimilarity > 4.2222 & slope structure = 0.6000 -> class stable [97.4%]
Rule 5: band1 > 62 & dem <= 75 & water level of reservoir = 0.1000 & slope structure = 0.6000 -> class stable [97.0%]
Rule 6: band4 > 155 & slope > 39.0801 & engineering rock group = 1.2000 & slope structure = 0.3000 -> class stable [97.0%]
Rule 7: band3secondmoment <= 0.1852 & engineering rock group = 0.4000 & slope <= 3.7242 & dem > 263 - > class stable [95.2%]
Rule 8: water level of reservoir = 0.1000 & band3variance > 11.8765 & slope structure = 0.6000 -> class stable [94.6%]
Rule 9: band3 <= 37 & dem <= 337 & slope structure = 0.3000 -> class stable [92.2%]
Rule10: band4 <= 128 & engineering rock group = 0.4000 & dem > 263 -> class stable [89.1%]
Rule 11: band3variance <= 11.8765 & dem > 75 & engineering rock group = 1.2000 & slope structure = 0.6000 -> class basically stable [99.5%]
Forecast criterions of basically stable region:
Rule1: dem <= 263 & engineering rock group = 0.4000 & slope structure = 1.5000 -> class basically stable [99.4%]
Rule2: band3variance <= 11.8765 & band3dissimilarity <= 4.2222 & band1 <= 62 & slope structure = 0.6000 -> class basically stable [99.4%]
Rule 3: water level of reservoir = 0.2000 & slope structure = 0.6000 -> class basically stable [99.3%]
Rule 4: NDVI > 0.2917 & band3 > 37 & band4 <= 155 & engineering rock group = 1.2000 & aspect <= 298.811 & slope structure = 0.3000 -> class basically stable [98.5%]
Rule 5: dem > 41 & dem <= 139 & water level of reservoir = 0.1000 & slope structure = 1.5000 -> class basically stable [98.4%]
Rule 6: NDVI > 0.2917 & band3 > 37 & slope <= 39.0801 & engineering rock group = 1.2000 & band4 <= 158 & slope structure = 0.3000 & aspect <= 298.811 -> class basically stable [98.0%]
Rule 7: engineering rock group = 1.2000 & slope structure = 0.9000 -> class basically stable [97.9%]
Rule 8: dem > 337 & engineering rock group = 1.2000 & slope structure = 0.3000 -> class basically stable [97.8%]
Rule 9: NDVI > 0.2917 & band3 > 37 & band4 <= 151 & engineering rock group = 1.2000 & slope structure = 0.3000 -> class basically stable [97.6%]
Rule 10: band2 <= 77 & engineering rock group = 1.2000 & slope structure = 1.5000 -> class basically stable [96.2%]
Rule 11: band3variance <= 1.284 & band4 > 128 & slope > 3.7242 & aspect <= 257.735 & engineering rock group = 0.4000 -> class basically stable [95.9%]
Rule 12: 3/2 <= 0.4949 & band3variance <= 1.284 -> class basically stable [93.2%]
Rule 13: band3variance > 8.1728 & dem <= 78 & engineering rock group = 1.2000 & slope structure = 1.5000 -> class basically stable [90.6%]
Rule 14: band4 > 150 & dem <= 126 & engineering rock group = 1.2000 & slope structure = 1.5000 -> class basically stable [89.1%]
Rule 15: engineering rock group = 1.2000 & water level of reservoir = 0.5000 & slope structure = 0.6000 -> class basically stable [87.1%]
Forecast criterions of unstable region:
Rule 1: engineering rock group = 2.0000 & slope structure = 0.3000 -> class unstable [99.5%]
Rule 2: NDVI <= 0.2917 & slope structure = 0.3000 -> class unstable [99.4%]
Rule 3: dem > 139 & engineering rock group = 1.2000 & slope structure = 1.5000 -> class unstable [99.3%]
Rule 4: dem > 78 & engineering rock group = 1.2000 & water level of reservoir = 0.2000 & slope structure = 1.5000 -> class unstable [99.2%]
Rule 5: dem <= 41 & engineering rock group = 1.2000 & slope structure = 1.5000 -> class unstable [98.8%]
Rule 6: dem > 362 & slope structure = 1.5000 -> class unstable [98.4%]
Rule 7: band3mean <= 20.7778 & band3 > 46 & dem <= 187 & slope structure = 1.5000 & aspect <= 259.38 & dem > 147 -> class unstable [97.8%]
Rule 8: band3 > 46 & band4 > 128 & slope structure = 1.5000 & aspect > 197.526 & aspect <= 259.38 & dem > 147 -> class unstable [97.1%]
Rule 9: band3mean > 19.6667 & band3homogeneity <= 0.3951 & band3contrast <= 19.2222 & dem <= 362 & engineering rock group = 2.0000 & aspect <= 197.526 & dem > 147 -> class unstable [96.5%]
Rule10: band3 > 46 & band4 > 145 & slope structure = 1.5000 & aspect <= 259.38 & dem > 147 -> class unstable [96.3%]
Rule11: band3homogeneity <= 0.5222 & band3correlation > −3.0981 & engineering rock group = 2.0000 & band3 > 46 & aspect <= 259.38 & dem > 147 & dem <= 362 -> class unstable [96.2%]
Rule 12: engineering rock group = 1.6000 -> class unstable [94.8%]
Rule13: band3mean > 20.7778 & band4 > 116 & water level of reservoir = 0.2000 & slope structure = 1.5000 & aspect > 197.526 & aspect <= 259.38 & dem > 147 -> class unstable [93.8%]
Rule 14: band3mean > 19.6667 & band3homogeneity <= 0.5222 & band3contrast <= 3.8889 & band4 <= 145 & aspect <= 197.526 & dem > 147 & dem <= 362 -> class unstable [93.7%]
Rule 15: band4 > 151 & band4 <= 158 & aspect > 298.811 & slope structure = 0.3000 -> class unstable [93.6%]
Rule 16: water level of reservoir = 0.4000 & band3mean <= 19.6667 & slope structure = 1.5000 & aspect <= 259.38 -> class unstable [93.6%]
Rule17: engineering rock group = 0.4000 & slope structure = 0.3000 -> class unstable [90.6%]
Rule 18: band3mean > 20.7778 & band3homogeneity > 0.304 & water level of reservoir = 0.2000 & slope structure = 1.5000 & band1 > 65 & aspect > 259.38 -> class unstable [87.9%]
Rule 19: water level of reservoir = 0.1000 & engineering rock group = 2.0000 & band4 <= 128 -> class unstable [82.0%]
Rule20: band3entropy > 2.0432 & engineering rock group = 2.0000 & slope structure = 1.5000 & band4 > 152 & aspect > 259.38 -> class unstable [75.8%]
Forecast criterions of dangerous region:
Rule 1: dem <= 147 & engineering rock group = 2.0000 & slope structure = 1.5000 -> class dangerous [97.9%]
Rule 2: engineering rock group = 2.0000 & slope structure = 0.9000 -> class dangerous [96.5%]
Rule 3: band4 <= 152 & engineering rock group = 2.0000 & slope structure = 1.5000 & aspect > 259.38 & dem <= 362 -> class dangerous [96.2%]
Rule 4: water level of reservoir = 0.4000 & engineering rock group = 2.0000 & slope structure = 1.5000 & band4 <= 128 -> class dangerous [95.0%]
Rule 5: band3mean <= 20.7778 & dem > 187 & engineering rock group = 2.0000 & band4 <= 128 -> class dangerous [94.8%]
Rule 6: band3homogeneity > 0.5222 & engineering rock group = 2.0000 & slope structure = 1.5000 & aspect <= 197.526 -> class dangerous [94.8%]
Rule 7: band3 <= 46 & engineering rock group = 2.0000 & aspect <= 259.38 & dem <= 362 -> class dangerous [94.4%]
Rule 8: engineering rock group = 2.0000 & slope structure = 0.6000 -> class dangerous [93.9%]
Rule 9: band3mean > 19.6667 & band3contrast > 19.2222 & engineering rock group = 2.0000 & slope structure = 1.5000 & dem <= 362 -> class dangerous [93.2%]
Rule10: band3mean <= 23.6667 & engineering rock group = 2.0000 & band4 <= 118 & aspect > 197.526 -> class dangerous [88.5%]
Rule 11: slope structure = 1.2000 -> class dangerous [88.2%]
Rule 12: band3mean <= 19.6667 & engineering rock group = 2.0000 & band3correlation <= −3.0981 & band4 <= 145 & aspect <= 197.526 & dem > 161 & dem <= 362 -> class dangerous [87.9%]
Rule 13: band3homogeneity > 0.3951 & band3contrast > 3.8889 & engineering rock group = 2.0000 & slope structure =1.5000 & band3correlation <= −3.0981 & aspect <= 197.526 & dem > 161 & dem <= 362 -> class dangerous [87.1%]
Rule 14: engineering rock group = 0.0000 -> class dangerous [63.0%]
Default Class: basically stable

**Table 3.** Connotation of the values of the forecast factors.

Values of forecast indexes	Connotation
slope structure=1.5	Slopes with the same direction
slope structure=0.6	Slopes with the converse direction
slope structure=0.3	Slopes with the horizontal direction
slope structure=0.9	Converse slope
slope structure=1.2	Direct slope
water level of reservoir=0.1	Poorly influenced region
water level of reservoir=0.2	Medium influenced region
water level of reservoir=0.4	Strongly influenced region
water level of reservoir=0.5	Fluctuating region
engineering rock group=1.2	Alternate soft and hard stratum
engineering rock group=1.6	Hard rock
engineering rock group=0.4	Soft rock
engineering rock group=2	Loose deposits

**Table 4.** Forecast precisions of decision tree and the other six ones.

**Precision assessment**	**Unsupervised method**		**Supervised method**				**Decision tree**
	**IsoData Method**	**K-Means Method**	**Paralle-lepiped**	**Minimum Distance**	**Mahalanobis Distance**	**Maximum likelihood**	
Overall Accuracy	15.99%	15.99%	46.59%	28.21%	73.44%	80.97 %	99.15%
Kappa Coefficient	0	0	0.2126	0.0960	0.6311	0.7322	0.9876
